# Exceptional Strength–Ductility Combinations of a CoCrNi-Based Medium-Entropy Alloy via Short/Medium-Time Annealing after Hot-Rolling

**DOI:** 10.3390/ma17194835

**Published:** 2024-09-30

**Authors:** Yongan Chen, Dazhao Li, Zhijie Yan, Shaobin Bai, Ruofei Xie, Jian Sheng, Jian Zhang, Shuai Li, Jinzhong Zhang

**Affiliations:** 1 School of Materials Science and Engineering, North University of China, Taiyuan 030051, China; cya894589913@163.com (Y.C.); yanzhijie74@163.com (Z.Y.); xieruofei2623@163.com (R.X.); shengjian032@163.com (J.S.); 2 Shanxi Key Laboratory of Advanced Metal Materials for Special Environments, Taiyuan 030051, China; 3 School of Aerospace Engineering, North University of China, Taiyuan 030051, China; sh1989bedu@163.com; 4 Department of Operation, Guoneng Huaian Co-Generation Co., Ltd., Zhangjiakou 076100, China; zjmtzs23h@163.com (J.Z.); 15830305563@163.com (S.L.); zm20001115@163.com (J.Z.)

**Keywords:** medium-entropy alloy, precipitates, mechanical properties, deformation mechanisms

## Abstract

Strong yet ductile alloys have long been desired for industrial applications to enhance structural reliability. This work produced two (CoCrNi)_93.5_Al_3_Ti_3_C_0.5_ medium-entropy alloys with exceptional strength–ductility combinations, via short/medium (3 min/30 min) annealing times after hot-rolling. Three types of intergranular precipitates including MC, M_23_C_6_ carbides, and L1_2_ phase were detected in both samples. Noticeably, the high-density of intragranular L1_2_ precipitates were only found in the medium-time annealed sample. Upon inspection of the deformed substructure, it was revealed that the plane slip is the dominant deformation mechanism of both alloys. This is related to the lower stacking fault energy, higher lattice friction induced by the C solute, and slip-plane softening caused by intragranular dense L1_2_ precipitates. Additionally, we noted that the stacking fault and twinning act as the mediated mechanisms in deformation of the short-time annealed alloy, while only the former mechanism was apparent in the medium-time annealed alloy. The inhibited twinning tendency can be attributed to the higher energy stacking faults and the increased critical twinning stress caused by intragranular dense L1_2_ precipitates. Our present findings provide not only guidance for optimizing the mechanical properties of high/medium-entropy alloys, but also a fundamental understanding of deformation mechanisms.

## 1. Introduction

As a class of advanced metal materials, high/medium-entropy alloys (H/MEAs) hold great promise for overcoming the property bottleneck in conventional alloys [1,2]. Among many studies [3,4,5], an equiatomic CrCoNi MEA with a face-centered cubic (FCC) structure has emerged as one of the most damage-tolerant alloys to date. Despite its excellent ductility, the alloy always showed a lower yield strength of approximately 350 MPa, because of the multiple slip systems, similar to other FCC alloys [6]. This severely limits its engineering serviceability for structural applications. Exploring appropriate strategies to overcome the strength–ductility trade-off has been a major objective for the CrCoNi MEA [7].

Many studies [8,9] have shown that the precipitation strengthening by a coherent L1_2_ phase may be an ideal method for enhancing the strength of FCC-structured H/MEAs without the excessive expense of ductility. For example, Zhao et al. [10] produced a L1_2_ phase-strengthened CoCrNi-based MEA by alloying Al and Ti elements, which exhibited a decent yield strength of 750 MPa with excellent plasticity of 45%. Wang et al. [11] reported a similar (CoCrNi)_94_Al_3_Ti_3_ MEA, strengthened by a L1_2_ phase, with a yield strength of 900 MPa and a fracture strain of 22.9%. In these studies [8,9,10,11], the L1_2_ phase usually co-exists in both intergranular and intragranular forms, as a result of discontinuous and continuous precipitations, respectively. It was revealed [10,11] that the intragranular L1_2_ generally precipitates at a high-density and nano-size, which not only enhances strength by hindering dislocation motion, but also allows the dislocations that shear through them to maintain plasticity.

Another effective strategy to enhance the FCC-H/MEAs by C-doping has also been widely reported [12,13]. In general, the interstitial C element plays a dual role in producing solid-solution strengthening and introducing carbides to trigger precipitation strengthening. Wang et al. [14] revealed in an FCC-HEA that the yield strength is linearly related to the C content with a specific growth rate of 184 MPa/at.%. Evidently, the effectiveness produced by solid-solution strengthening is relatively limited. In comparison, the carbides generally play a dominant strengthening role for improving the mechanical properties of H/MEAs [13]. Moravcik et al. [15] reported in a C-doping CoCrNi MEA where the introduction of M_23_C_6_ carbides enabled the yield strength to increase from 335 MPa to 595 MPa, while maintaining an excellent plasticity of 62%. Interestingly, in addition to the strengthening effect, it was shown that the carbides can also play a constructor role for the grain architecture [16]. Specifically, during the annealing phase, the rapid precipitation of carbides at grain boundaries generally occurs in conjunction with recrystallization; the carbides could promote recrystallization by providing favored nucleation sites at the initial annealing stage; however, the uniform extension of grain boundaries at later annealing stages is inhibited because of the Zenner-pinning effects induced by carbides. This generally contributes to a fine-grained or hetero-grained microstructure in annealed alloys [12,16] that could avoid the possible loss of strength due to grain coarsening.

Inspired by the above studies, we designed a (CoCrNi)_93.5_Al_3_Ti_3_C_0.5_ MEA for employing a composite precipitation–strengthening strategy of L1_2_ phase and carbides to enhance the strength–ductility synergy. A coupled production process of hot-rolling and annealing was adopted to construct the microstructure. In order to clearly observe the microstructural evolution, short-time annealing and medium-time annealing were carried out separately for comparison purposes. The interaction behaviors of multiple precipitations and their effect on grain structure evolution were focused on. Also, the deformation mechanisms of the alloys were revealed to gain an insight into the connection between the microstructure and mechanical properties.

## 2. Materials and Methods

The investigated MEA with a nominal composition of (CoCrNi)_93.5_Al_3_Ti_3_C_0.5_ (at.%) was prepared by vacuum induction melting with pure elements (>99.9 purity). After drop-casting, the ingot, which weighed about 50 kg, was homogenized at 1200 °C for 4 h and then hot-rolled at 1100 °C from 60 mm to 3 mm (a thickness reduction ratio of ~95%). Subsequently, the as-rolled sheet was isothermally annealed at 950 °C for 3 min and 30 min, respectively. Any of the thermomechanical treatments were conducted in air and finished by water-quenching. For convenience, the annealed samples were code-named HA-3 and HA-30, respectively.

Quasi-static tensile tests were performed on a universal electronic tensile testing machine (AG-X PLUS) at room temperature. The dog-bone specimens were fabricated with a gauge length of 25 mm and a cross-section area of 6 × 3 mm^2^. Tensile loading was parallel to the rolling direction, with a strain rate of 1 × 10^−3^ s^−1^. At least three repeated tests were conducted for authenticity. An X-ray diffractometer (XRD, SmartLab-3 KW) was employed for phase identification, with an operating range from 20 to 90 deg. Microstructural observations were conducted by a SIGMA500 field-emission scanning electron microscope (SEM) equipped with an Oxford Nordlys Max3 for electron backscattered diffraction (EBSD), and a Tecnai G2 F30 S-TWIN transmission electron microscope (TEM) equipped with energy-dispersive X-ray spectroscopes (EDS). The EBSD examination was performed with a step size of 0.05 μm. Both the SEM and EBSD specimens were taken along the rolled direction. After mechanically grounding and polishing, the specimens were electro-polished (15 V, 273 K) with an electrolyte composed of 90% ethanol and 10% perchloric acid (vol.%). The specimens for TEM analysis were mechanically thinned to ~50 µm and punched into 3 mm diameter foils, followed by ion-beam thinning at ambient temperature.

## 3. Results and Discussion

### 3.1. Phase Composition and Grain Structure

**Figure 1:**
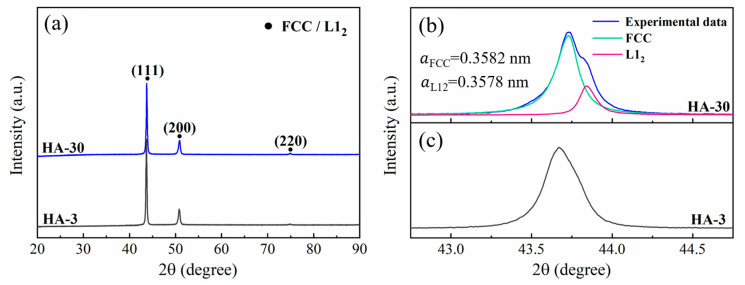
(**a**) XRD patterns of the HA-3 and HA-30 samples. Deconvolution (**b**) and local amplification (**c**) of the (111) diffraction peak for the HA-30 and HA-3 samples, respectively.

The XRD patterns of the studied samples are shown in Figure 1a. Only the typical diffraction peaks representative of the FCC matrix can be detected. Of note, the peaks of the HA-30 sample show a little asymmetry, generally indicating the presence of an ordered L1_2_ phase [17]. Through deconvolving the (111) diffraction peak (Figure 1b), the lattice constants of the FCC and L1_2_ phases were calculated to be 0.3582 nm and 0.3578 nm, respectively. The phenomenon of peak overlap is not obvious in the HA-3 sample (Figure 1c), suggesting that the presence of L1_2_ phase needs to be further verified.

**Figure 2:**
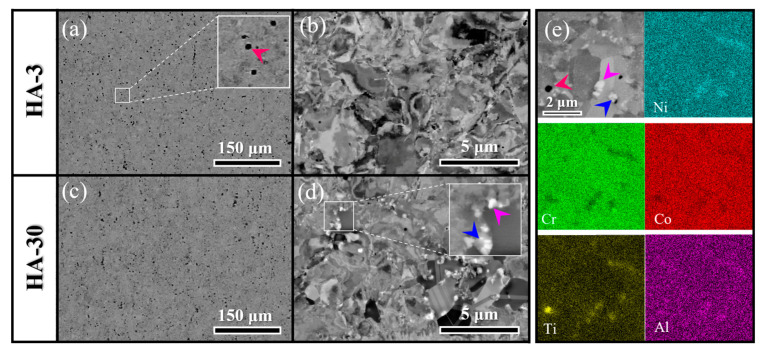
BSE images showing the microstructures of (**a**,**b**) HA-3 and (**c**,**d**) HA-30 samples. (**e**) Elemental distributions of the precipitates present in the alloy studied.

The backscatter electron (BSE) technique was employed to examine the microstructure. From the low-magnification BSE images (Figure 2a,b), it is observed that the black particles are uniformly distributed in both samples. Upon magnification, some fine particles distributed at grain boundaries (GBs) can be found in the HA-3 sample (Figure 2c). With the annealing time prolonged to 30 min (HA-30), the intergranular particles grew and could be clearly identified as grey and white (Figure 2d). As shown in Figure 2e, the SEM-EDS mapping results reveal that the black particles are enriched with the element, Ti, while the grey and white ones are enriched with Cr and the elements Ni, Al, and Ti, respectively, (C was excluded because of the limited precision of the instrument).

**Figure 3:**
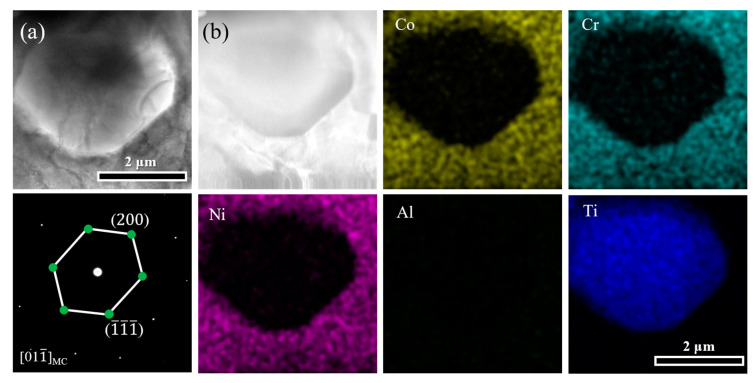
(**a**) The BF image showing the MC carbide confirmed by the corresponding SAED pattern. (**b**) Elemental distribution of the MC carbide.

In order to identify the precipitates accurately, detailed microstructure analyses of the HA-30 sample based on TEM technology were carried out. The bright-field (BF) image in Figure 3a shows a micron-sized particle, which can be identified as MC carbide by selected-area electron diffraction (SAED). The corresponding TEM-EDS mapping (Figure 3b) reveals that the MC carbide is rich in Ti, and depleted in Co, Cr, Ni, and Al, consistent with the black particles in the BSE results. It has been reported that the higher affinity and negative mixing enthalpies of C–Ti atom pairs is favorable for the formation of MC carbides [18]. According to the calculated phase diagram in our previous study [7], it is speculated that the MC carbides were mainly inherited from the solidification phase and grow further during the solid solution and hot rolling stages, thus reaching sizes on the micrometer-scale.

**Figure 4:**
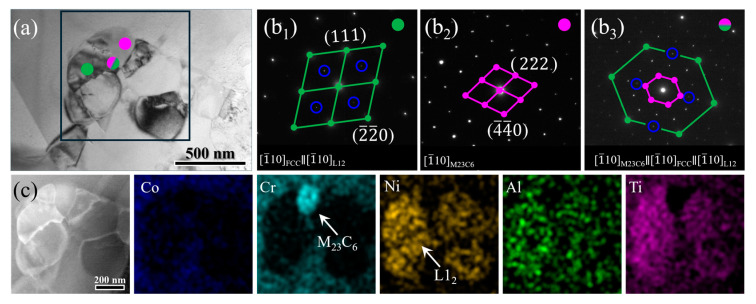
(**a**) The BF image showing a cluster of precipitates, which were identified as M_23_C_6_ carbides and L1_2_ phase, respectively, by the corresponding SAED patterns (**b1**–**b3**). (**c**) Elemental distributions of the M_23_C_6_ carbides and L1_2_ phase.

Figure 4a shows a cluster of precipitates distributed in the GB regions. Through combining the SAED (Figure 4b_1_,b_2_) and TEM-EDS mapping (Figure 4c), two types of precipitates of M_23_C_6_ carbides and L1_2_ phase were identified. The former is enriched with Cr, while the latter with Ni, Al, Ti, which is consistent with the grey and white particles in the BSE results, respectively. Upon inspection of many BSE and BF-TEM images, it is noted that the M_23_C_6_ carbide and L1_2_ phase were almost always distributed adjacently. Moreover, we found that the M_23_C_6_ carbide, FCC matrix, and L1_2_ phase show a cubic–cubic orientation relationship, i.e., [110]_M23C6_∥[110]_FCC_∥[110]_L12_ (Figure 4b_3_). These observations may indicate an interactive correlation between the formation of the M_23_C_6_ carbide and L1_2_ phase. Specifically, the rapid diffusion of C atoms at high temperatures favors the preferential precipitation of M_23_C_6_ carbides at GB regions; this can not only deplete Cr to create in turn a favorable elemental environment locally enriched in Ni, Al, and Ti, but also provide favorable nucleation sites, thus contributing to the precipitation of the L1_2_ phase adjacent to the M_23_C_6_ carbides.

**Figure 5:**
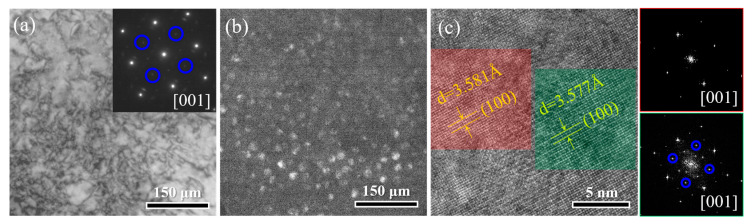
The TEM observations show the intragranular L1_2_ precipitates of the HA-30 sample. (**a**) BF and SAED images. (**b**) DF pattern. (**c**) HRTEM and corresponding FFT images.

Previous reports [19,20] have shown that in similar Al/Ti-doping 3d transition H/MEAs, the nano-scaled L1_2_ particles are typically precipitated in the grain interior uniformly upon medium-temperature annealing. Therefore, we conducted a further observation for the intragranular microstructure of both the samples studied. Figure 5a is a BF image of the HA-30 sample, showing the residual dislocations. The SAED pattern shows the weak spots in addition to the main diffraction spots representative of the FCC matrix, indicative of the presence of an ordered L1_2_ phase. The dark-field (DF) image in Figure 5b shows the diffuse distribution of nano-scaled L1_2_ particles. Figure 5c is the high-resolution (HR) TEM image and corresponding Fast Fourier transform (FFT) images. By measuring the crystal plane spacing directly, the lattice constants of FCC and L1_2_ phases were calculated to be 0.3581 nm and 0.3577 nm, respectively, almost in agreement with that from the XRD result. Inserting the values into the equation of $\delta =2\left(a_{{L1}_{2}}-a_{FCC}\right)/\left(a_{{L1}_{2}}+a_{FCC}\right)$ yielded the lattice mismatch value (δ) of 0.11%. Such a low lattice mismatch contributed to a lower elastic-misfit energy barrier for high-density nucleation [21]. Meanwhile, the sluggish diffusion rates of solutes in the multicomponent matrix and the lower interfacial energy (coherent interfaces of FCC/L1_2_ phases) suppressed the kinetic and thermodynamic driving forces of Ostwald ripening, respectively [22,23]. These were jointly responsible for the high-density yet nanoscale of the intragranular L1_2_ precipitates. In contrast to the HA-30 sample, almost none of the intragranular L1_2_ precipitates were detected in the HA-3 sample, probably due to the very short annealing time. This rationalizes the phenomenon of insignificant peak overlap in the XRD pattern.

**Figure 6:**
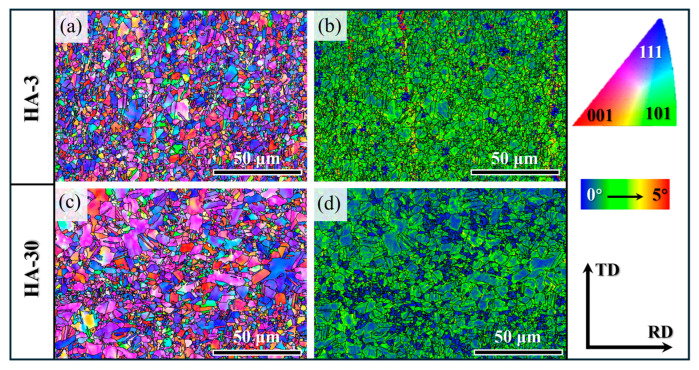
IPF (**a**,**c**) and KAM (**b**,**d**) images of the HA-3 and HA-30 samples, respectively. TD: transverse direction; RD: rolling direction.

EBSD examinations were conducted to uncover the recrystallization microstructure. As shown in Figure 6a,c, the inverse pole figure (IPF) maps of the present samples both show the {111} texture orientation, corresponding to the orientation peak amplitude of the (111) plane in the XRD patterns. The HA-3 sample presented a relatively homogenous grain structure consisting of uniformly sized equiaxed-grains (Figure 6a). The average grain size was evaluated to be 3.48 ± 0.9 μm, which is close to that of the hot-rolled samples (3.50 ± 0.9 μm, as shown in Figure S1a). Corresponding kernel average misorientation (KAM) distribution mapping (Figure 6b) shows a high density of residual dislocations almost throughout the microstructure, like in the hot-rolled sample (Figure S1b), indicative of the lower degree of recrystallization. As the annealing time increased to 30 min, the density of the residual dislocations decreased significantly, while part of the grains was coarsened and some recrystallized fine-grains formed (Figure 6c,d). The average grain size was increased to 4.64 ± 0.9 μm. Compared to the CoCrNi MEA [24], the degree of grain coarsening caused by annealing is relatively slight in the studied alloy. This is attributed to the joint effects of solute dragging and precipitate-induced Zener pinning [16]. Additionally, with regard to the heterogeneous grain structure of the HA-30 sample, besides the possibly uneven distribution of deformation storage energy, the effect of intergranular precipitation behavior may be the main reason for the inhomogeneous recrystallization [24]. On the one hand, the particles preferentially precipitated at the GB regions provide heterogeneous nucleation sites for recrystallization. Or, the intergranular precipitates produced Zener pinning forces that inhibit the homogeneous expansion of GBs.

### 3.2. Mechanical Properties

**Figure 7:**
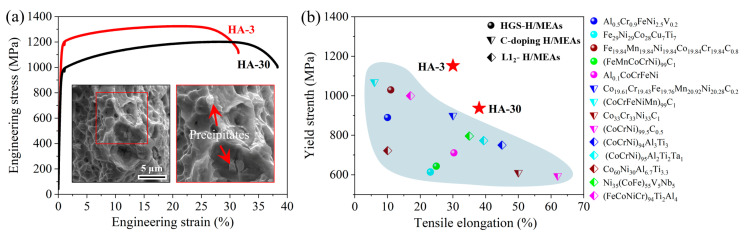
(**a**) Engineering stress–strain curves of the studied alloy and the fracture morphology of the HA-30 sample. (**b**) Comparison of strength–ductility between the studied samples with other similar H/MEAs [12,16,19,20,25,26,27,28,29,30,31,32,33].

The engineering stress–strain curves of the present samples are shown in Figure 7a. The HA-3 sample exhibited a yield strength (YS) of 1152 MPa, an ultimate tensile strength (UTS) of 1324 MPa, and a total elongation (TE) of 30%. In comparison, the YS and UTS of the HA-30 sample decreased to 937 MPa and 1202 MPa, respectively, while the TE increased to 38%. It can be inferred that the decreased YS was due to the decrease in dislocation density, the increase in grain sizes, and the coarsening of intergranular precipitates. However, the additional precipitate strengthening from the intragranular L1_2_ particles and the hetero-deformation induced (HDI) stress strengthening provided some compensation for the strength. Thus, the reduction of YS was not severe. In addition, it is noted that although the HDI effect was conducive to enhancing the work-hardening response, the local stress concentration at coarsened intergranular precipitates may be a source that leads to premature crack initiation (as shown in the insert in Figure 7a). Therefore, the increase of TF was not significant. Figure 7b shows the exceptional strength–ductility combination of the current samples, which is superior to most of other similar H/MEAs [12,16,19,20,25,26,27,28,29,30,31,32,33]. This indicates the effectiveness of the strengthening–toughening strategy in this work for optimizing the mechanical properties.

### 3.3. Deformation Mechanisms

**Figure 8:**
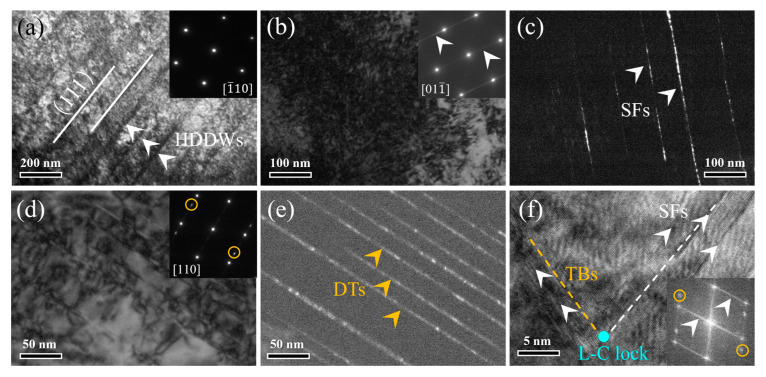
TEM observations of the deformed substructures of the HA-3 sample. (**a**) The BF image showing the (111) slip plane and HDDWs. (**b**,**c**) The SFs shown in the BF and DF images, respectively. (**d**,**e**) The DTs shown in the BF and DF images, respectively. (**f**) The HRTEM image showing the L–C lock formed by the interaction of SFs and TBs.

Detailed deformation substructures were examined via TEM analyses of the fractured samples to reveal the deformation mechanisms. As shown in Figure 8a, the typical (111) plane slip trace and the high-density dislocation walls (HDDWs) can be observed in the HA-3 sample. This indicates the planar slip mechanism, generally prevailed in FCC alloys with a lower stacking fault energy (SFE) [10]. Figure 8b shows the dislocation tangles in a BF image, where the inserted SAED pattern presents the streaky lines. The deformation-induced stacking faults (SFs) are clearly shown in the corresponding DF image, as marked in Figure 8c. Figure 8d,e show the deformation twins (DTs) in the BF and DF images, respectively. The twin-related spots were marked in the corresponding SAED pattern (as shown in the insert in Figure 8d) taken along the [110] zone axis. The HRTEM image shows the Lomer–Cottrell (L–C) lock that was formed by the interactions of SFs and twin boundaries (TBs), as marked in Figure 8f. The corresponding FFT images shown in the inserts present both the streaky lines and twin-related spots.

**Figure 9:**
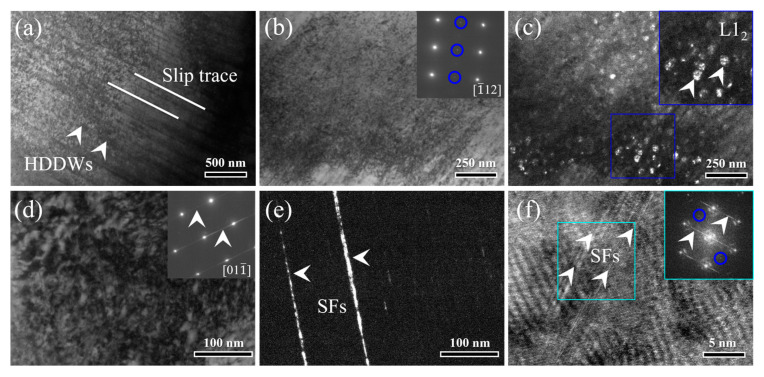
TEM observations of the deformed substructures of the HA-30 sample. (**a**) The BF image showing the parallel slip plane and HDDWs. (**b**,**c**) The L1_2_ precipitates after deformation shown in the BF and DF images, respectively. (**d**,**e**) The SFs shown in the BF and DF images, respectively. (**f**) The SFs shown in the HRTEM image, and the coexistence of streaky lines and weak spots shown in the inserted FFT pattern.

Similar to the HA-3 sample, the parallel plane slip trace and HDDWs substructures were also observed in the HA-30 sample (Figure 9a). A detailed examination of the slip region revealed almost non-existent dislocation pile-up or dislocation loop substructures, although the inserted SAED pattern indicated the presence of L1_2_ precipitates (Figure 9b). From the DF image (Figure 9c), it appears that new interfaces were produced in the L1_2_ precipitates after deformation, making their shapes irregular. Previous reports [11,25,26] have shown that the slip dislocations usually shear rather than bypass the coherent L1_2_ precipitates because the coherent phase interfaces have a lower lattice misfit. This is effective in avoiding dislocation accumulation and thus the concentration of local stress points. Figure 9d,e show the SFs in the BF and DF images, respectively. The SFs were further characterized through the HRTEM technology, as shown in Figure 9f. The inserted FFT pattern taken from the marked region presents both the streaky lines and faint superlattice reflection spots. This indicates that the SFs can also shear the L1_2_ precipitates. Noticeably, in contrast to the HA-3 sample, none of the DTs were detected upon the detailed SAED examinations for the HA-30 sample.

Based on the above observations, it was revealed that the deformation of both the HA-3 and HA-30 samples is mainly dominated by the plane slip mechanism. One major difference is that the SFs and DTs play a mediated role in the former, while only the SFs affect the latter. Previous reports [4] have shown that a lower SFE can facilitate the full dislocations that relate to plane slip or dissociate into two Shockley partial dislocations, reducing the propensity of cross slip. Referring to other studies [10,11], the SFEs of equiatomic CoCrNi-based MEAs have generally been evaluated as 20~30 mJ/m^2^. It is predicted that the present (CoCrNi)_93.5_Al_3_Ti_3_C_0.5_ MEA discussed in this work also has a low SFE, contributing to the plane slip mechanism. In addition, it has been shown that the solute of the element C can also promote the plane slip mechanism, as the enhanced lattice friction results in the slowing of the dislocation movement [12]. For the HA-30 sample, there was an additional factor, i.e., the high density of intergranular L1_2_ precipitates that induce slip plane softening, contributing to the plane slip mechanism [34]. Specifically, the L1_2_ precipitates act as obstacles to dislocation slip that is energetically favorable for the formation of dislocation pairs [35]. As the first dislocation shears through the L1_2_ ordered domain, high-energy bonds will be produced that trigger the increased system energy; with the entry of the second dislocation, with the same Burgers vector as the first one, the bonding within the ordered region will be restored and thus the system energy reduced, so it is energetically favorable [36]. After several dislocation pairs shear through the L1_2_ ordered domain, the resistance to dislocation movement will be reduced, and the subsequent shearing will not need the slip of dislocation pairs. As a result, the slip planes will be softened, promoting the plane slip mechanism.

Another point worth noting is that the absence of DTs in the HA-30 sample is also related to the intergranular L1_2_ precipitates. It is established that the formation of DTs is closely associated with the extension behavior of the SFs. Previous studies [37] have reported that when the SFs shear the ordered L1_2_ precipitates, complex SFs (CSFs) with higher energy will be produced as a result. Eliminating the CSFs’ ability to form twins requires diffusion-mediated reordering within the L1_2_ ordered domain, as revealed in the Ni-based superalloys [38]. However, this was apparently almost impossible to achieve at room temperature, and thus the formation of DTs was inhibited. In addition, it has been shown that the high density of L1_2_ precipitates narrowing the matrix channels and thus enhancing the critical twinning stress ($\tau_{tw}$) is also an important factor in suppressing the twinning behavior. The corresponding theorical equation is expressed below [10,36]:

(1) $$\tau_{tw}=\frac{2\alpha Gb_{p}}{L}+\frac{\gamma_{ISFE}}{b_{p}}+\tau_{fr}$$

(2) $$L=\sqrt{\frac{8}{3\pi f}}d-d$$

where α is a constant reflecting the dislocation character (0.5 [36]), G is the shear modulus (88.7 GPa [37]), $b_{p}=\frac{a}{\sqrt{6}}=0.146\;\mathrm{nm}$ (measured from the XRD result) is the Burgers vector of Shockley partial dislocations, $\gamma_{ISFE}\;$ is the stacking fault energy (~22 mJ/m^2^ [10], taken from the value of CoCrNi MEA for simplicity), $\tau_{fr}$ is the lattice friction stress (51 MPa [37]), L is the effective length of a twinning source, f=8% and d=11 nm represent the volume fraction and average diameter of the intragranular L1_2_ precipitates (measured on average from multiple DF-TEM images). The critical shear stress and normal stress for twinning in the HA-30 sample were calculated to be 724 MPa and 2413 MPa, respectively. The latter is far higher than the sample’s UTS (1070 MPa), which can rationalize the absence of DTs upon deformation to fracture.

## 4. Conclusions

Two strong yet ductile MEAs with a composition of (CoCrNi)_93.5_Al_3_Ti_3_C_0.5_ were produced by short/medium-time annealing after hot-rolling. The microstructural evolution involving multiple precipitations and their effect on recrystallization grain structure during annealing was uncovered. Also, the deformation mechanisms of the two samples related to SFE, solute elements, and precipitates were revealed. The intragranular L1_2_ phase is the main factor responsible for the different deformation mechanisms between the studies of the two samples. This work provides not only an effective strategy for optimizing the mechanical properties but also a fundamental understanding of the deformation mechanisms of FCC-H/MEAs. The main conclusions can be summarized as follows:

1. Exceptional strength–ductility combinations were shown in both the HA-3 and HA-30 samples, with a yield strength of 1152 MPa and 937 MPa, ultimate tensile strength of 1324 MPa and 1202 MPa, and total elongation of 30% and 38%, respectively.
2. Three kinds of intergranular precipitates including MC, M_23_C_6_ carbides, and L1_2_ phase were detected in both the HA-3 and HA-30 samples. Note that the latter two precipitates are always distributed adjacently. This is because the preferentially formed M_23_C_6_ carbides provide favorable nucleation sites and a complementary elemental environment for L1_2_ precipitation.
3. The deformation of the HA-3 and HA-30 samples was dominated by the plane slip mechanism, with the former being mediated by SFs and DTs, while the latter was mediated by SFs only. The absence of DTs in the HA-30 sample was related to the high-density of intragranular L1_2_ precipitates which were absent in the HA-3 sample.

## Data Availability

Data will be made available upon request.

## Supplementary Materials

The following supporting information can be downloaded at: https://www.mdpi.com/article/10.3390/ma17194835/s1, Figure S1: IPF (a) and KAM (b) images of the hot-rolled samples. TD: transverse direction; RD: rolling direction.
