# Supplementary material for: Exceptional Strength–Ductility Combinations of a CoCrNi-Based Medium-Entropy Alloy via Short/Medium-Time Annealing after Hot-Rolling
Source: Materials (Basel). 2024 Sep 30;17(19):4835. doi: 10.3390/ma17194835 (PMC11478033; doi:10.3390/ma17194835)
Supplement: Supplementary file 1 [file materials-17-04835-s001.zip › materials-3184123-supplementary.pdf]

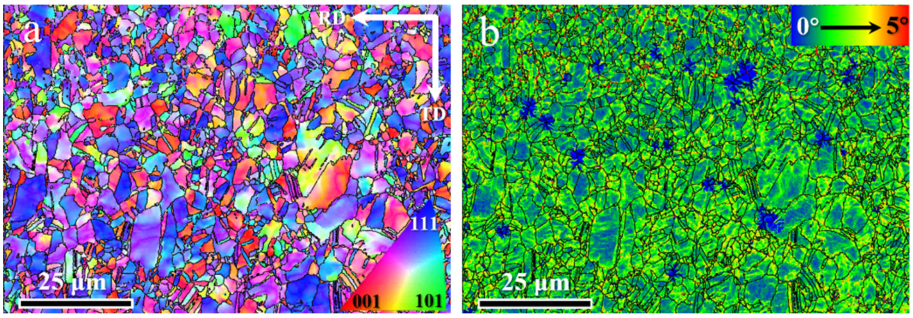

Figure S1. IPF (**a**) and KAM (**b**) images of the hot-rolled samples. TD: transverse direction; RD: rolling direction.
